# Vaccination with parasite-specific TcTASV proteins combined with recombinant baculovirus as a delivery platform protects against acute and chronic *Trypanosoma cruzi* infection

**DOI:** 10.3389/fcimb.2024.1297321

**Published:** 2024-02-28

**Authors:** Yamil E. Masip, Lucas D. Caeiro, Maximiliano Cosenza, Miriam Postan, Guido Molina, Oscar Taboga, María Paula Molinari, Valeria Tekiel

**Affiliations:** ^1^ Instituto de Investigaciones Biotecnológicas, – Consejo Nacional de Investigaciones Científicas y Técnicas (CONICET), Buenos Aires, Argentina; ^2^ Escuela de Bio y Nanotecnologías (EByN), Universidad Nacional de San Martín (UNSAM), Buenos Aires, Argentina; ^3^ Consejo Nacional de Investigaciones Científicas y Técnicas (CONICET), Buenos Aires, Argentina; ^4^ Instituto de Agrobiotecnología y Biología Molecular (IABIMO), Instituto Nacional de Tecnología Agropecuaria (INTA), Consejo Nacional de Investigaciones Científicas y Técnicas (CONICET), Buenos Aires, Argentina

**Keywords:** Chagas’ disease, vaccine, trypomastigote, multigene family, TcTASV, baculovirus

## Abstract

Chagas’ is a neglected disease caused by the eukaryotic kinetoplastid parasite, *Trypanosoma cruzi.* Currently, approximately 8 million people are infected worldwide, most of whom are in the chronic phase of the disease, which involves cardiac, digestive, or neurologic manifestations. There is an urgent need for a vaccine because treatments are only effective in the initial phase of infection, which is generally underdiagnosed. The selection and combination of antigens, adjuvants, and delivery platforms for vaccine formulations should be designed to trigger mixed humoral and cellular immune responses, considering that *T. cruzi* has a complex life cycle with both intracellular and bloodstream circulating parasite stages in vertebrate hosts. Here, we report the effectiveness of vaccination with a *T. cruzi*-specific protein family (TcTASV), employing both recombinant proteins with aluminum hydroxide and a recombinant baculovirus displaying a TcTASV antigen at the capsid. Vaccination stimulated immunological responses by producing lytic antibodies and antigen-specific CD4+ and CD8+ IFNɣ secreting lymphocytes. More than 90% of vaccinated animals survived after lethal challenges with *T. cruzi*, whereas all control mice died before 30 days post-infection. Vaccination also induced a strong decrease in chronic tissue parasitism and generated immunological memory that allowed vaccinated and infected animals to control both the reactivation of the infection after immunosuppression and a second challenge with *T. cruzi*. Interestingly, inoculation with *wild-type* baculovirus partially protected the mice against *T. cruzi*. In brief, we demonstrated for the first time that the combination of the baculovirus platform and the TcTASV family provides effective protection against *Trypanosoma cruz*i, which is a promising vaccine for Chagas disease.

## 1 Introduction

Chagas’ disease is a zoonotic infection caused by the kinetoplastid parasite, *Trypanosoma cruzi*. While its primary transmission route involves triatomine insects, which are endemic in the Americas, non-vectorial transmission, such as through blood transfusions and congenital transmission, has facilitated the spread of the disease worldwide. It is estimated that 6-8 million people are infected. (World Health Organization, 2023). The infection is characterized by a usually asymptomatic short acute phase followed by a chronic phase, where 30 to 40% of patients will eventually develop organ pathologies such as digestive alterations, cardiac failure, and death, 10 or more years after the initial infection (Pérez-Molina and Molina, 2018). Currently available treatments are effective only during the acute phase and have severe side effects (Sales Junior et al., 2017). Moreover, there is no preventive or therapeutic vaccine for *T. cruzi* infection. Ideally, a vaccine to prevent *T. cruzi* infection should confer protection against both parasite stages in the mammalian host: trypomastigotes and amastigotes. Trypomastigotes are the bloodstream, extracellular, and infective stage, and their presence in circulation is the hallmark of the acute phase. During this phase, the trypomastigotes spread infection to various organs and tissues. This process involves several rounds of cell invasion, intracellular replication as amastigotes, differentiation into trypomastigotes, cell lysis, and release of trypomastigotes into the intercellular space. Trypomastigotes may then infect neighboring cells or spread to distant tissues via circulation. Acute infection is controlled by the immune system a few months after the initial infection. Thereupon parasites are no longer found in circulation but remain persistently in tissues as intracellular amastigotes in the chronic phase of the infection. Therefore, the immune response naturally triggered by infection is sufficient to control parasitemia but does not completely eliminate the parasite (Acevedo et al., 2018). The precise immune mechanisms involved in the protection of the host against *T. cruzi* are still unclear, but several studies have demonstrated that an effective immune response should have a Th1 profile, including both humoral and cellular responses, with gamma interferon (IFNɣ) as the major cytokine promoting the activity of phagocytes, T helper cells (CD4+), and cytotoxic T lymphocytes (CD8+) (Rios et al., 2019). Furthermore, despite the incomplete understanding of the shape of a fully protective immunity against *T. cruzi*, there are key cells and components of the innate immune response (macrophages, DCs, NK, TLRs, NODs, and cytokines) that should be activated to favor an adequate immune profile that eventually leads to an anti-*T. cruzi* response (Cerbán et al., 2020; Gil-Jaramillo et al., 2021). All these immune-mediated processes should be considered in the rational design of anti-*T. cruzi* vaccine. Another factor to be taken into account for the development of an anti-*T. cruzi* vaccine is the adequate selection of antigens, which should be conserved among the different lineages of the parasite, expressed in the mammalian stages, and do not possess immunodominant epitopes.

*T. cruzi* Trypomastigote Alanine, Serine, and Valine-rich (TcTASV) is a multigene family that is highly conserved among lineages and lacks orthologs in other organisms, including trypanosomatids. TcTASV has approximately 40 members that can be classified into subfamilies based on their sequence similarity. All TcTASV subfamilies are expressed in trypomastigotes, but there are differences between their subcellular locations and their additional expression in other parasite stages (García et al., 2010; Bernabó et al., 2013). In particular, TcTASV-A and TcTASV-C are the most abundant subfamilies, have the highest levels of expression, and are in contact with the host immune system *in vivo* (Bernabó et al., 2013; Brunoro et al., 2015; Floridia-Yapur et al., 2016, 2019; Caeiro et al., 2018). The TcTASV-C subfamily is solely expressed in trypomastigotes, located at the parasite surface, and secreted both freely and contained in extracellular vesicles (Bernabó et al., 2013; Caeiro et al., 2018). Previously, a pool of protective antigens was identified using a cDNA expression library immunization approach, among which there was a fragment of a TcTASV-C gene (Tekiel et al., 2009). Thereafter, TcTASV-C was assessed as a vaccine in prime (DNA)- boost (protein) schemes and as a recombinant protein (rTcTASV-C) in combination with different adjuvants (Caeiro et al., 2018, 2020). Delays in the emergence of circulating parasites and the presence of functional antibodies involved in complement-mediated lysis have been systematically reported, strongly suggesting that the anti-TcTASV-C response is involved in controlling the early acute phase of infection. In addition, these immunization schemes yielded 30-40% survival rates after challenge with a highly virulent *T. cruzi* strain (0% survival in the control group). Although vaccination with TcTASV-C provided partial protection against *T. cruzi*, its performance could be improved by the incorporation of additional antigens. We reasoned that a protein expressed at the amastigote stage, together with a novel delivery system and adjuvant, aimed at strengthening the cellular immune response, could improve vaccination efficiency, particularly the survival rates of vaccinated animals.

Baculovirus (BV) are lepidopteran insect viruses widely used to express recombinant proteins in insect cells for various applications, including vaccines (Felberbaum, 2015; Sullivan et al., 2022). BVs can also be employed as delivery platforms for gene therapy, because they do not replicate in mammals. BVs have also been employed in immunotherapies, for example in malignancies such as cancer, because of their ability to induce strong innate immune responses with production of proinflammatory cytokines and type I interferon (Abe and Matsuura, 2010; Luo et al., 2011; Kondou et al., 2019; Targovnik et al., 2021). Another less-explored application of BV is as recombinant vaccine delivery platforms, displaying heterologous antigens at the surface or capsid (Premanand et al., 2018; Yoon et al., 2021). Presentation of the antigen at the capsid or envelope surface of recombinant BV is crucial because differential immune responses are triggered (Tavarone et al., 2017). In particular, we have previously demonstrated that the expression and presentation of a model antigen at the BV capsid promotes strong cellular responses, along with a specific IFNɣ production by cytotoxic CD8+ lymphocytes (Molinari et al., 2011, 2022).

In this study, we present the selection of an additional vaccine candidate belonging to the TcTASV-A subfamily and a vaccination scheme that combines rTcTASV-C and a recombinant BV displaying a fragment of a TcTASV-A protein at the capsid. This resulted in strong protection of immunized animals when challenged with lethal doses of *T. cruzi*, showing more than 90% survival and a reduction in parasitic load in tissues of more than 95%.

## 2 Methods

### 2.1 Ethical statement, mice and parasites

C3H/He and BALB/c mice were bred and maintained at the animal facilities of Instituto de Investigaciones Biotecnológicas (UNSAM-CONICET), in accordance with norms of the Comité Institucional para el Cuidado y Uso de Animales de Experimentación (CICUAE) of UNSAM. All vaccination and challenge experiments were approved by the protocols n° 01/2018, 07/2018, 08/2018, and 09/2019 (CICUAE).

Vero cells were grown at 37°C with 5% CO_2_ in MEM (Gibco) supplemented with 4% fetal bovine serum (FBS; Natocor), 100 U/mL penicillin (Sigma), and 10 μg/mL streptomycin (Sigma). For *in vitro* assays, culture-derived trypomastigotes were obtained from infected Vero cells supernatants, as previously described (Bernabó et al., 2013). Alternatively, Vero cells were grown on coverslips on p24 plates, infected with trypomastigotes, and cells containing amastigotes were fixed and processed by immunofluorescence at 96 h post-infection.

Bloodstream trypomastigotes were maintained by weekly passaging in CF1 mice with 10^5^ trypomastigotes at the BLS3 laboratory at UNSAM. For *in vivo* assays, bloodstream trypomastigotes, were obtained at the peak of parasitemia, counted on a Neubauer chamber, diluted in PBS-1% BSA, and employed to challenge vaccinated or control mice (Caeiro et al., 2020). The parasites used were from the RA (TcVI) strain (González Cappa et al., 1981).

### 2.2 Recombinant proteins and antisera

rTcTASV-C (amino acids 64 to 319 of GenBank CBI68067.1, which corresponds to the RA TcTASV-C gene FN599132.1) cloned in pGEX-3X was expressed and purified as previously described (Bernabó et al., 2013; Caeiro et al., 2018, 2020). This TcTASV-C is identical to C3747_32g182 (TCC strain, TcVI) and Tcruzi_1863-4-1211-93, a CL Brener ORF (unfortunately, ORFs are no longer available in TriTrypDB), and presented 94% identity with other TcTASV-C proteins. rGST, the fusion protein tag of rTcTASV-C, was obtained following the same procedure.

rTcTASV-A was produced by cloning an internal TcTASV-A fragment (amino acids 27–173 of TcCLB.506573.5) into the pGEM-T Easy vector. Adequate cloning was verified by sequencing, and this region of TcTASV-A was recloned into a pBAD vector in frame with GST and eight histidines (8xHis) to obtain the desired construct (Bouvier et al., 2018). rTcTASV-A was expressed in *E. coli* BL21, purified by affinity to Ni NTA agarose (Qiagen), and eluted with Imidazole (80-160 mM) using standard methods. Recombinant proteins were dialyzed against PBS after purification, quantified by Bradford assay, and stored (aliquoted) at -80°C. Additionally, proteins that were employed for mouse immunization and *in vitro* stimulation of splenocytes were treated with Polymyxin B (Detoxi-Gel Endotoxin Removing Gel Thermo Scientific) in a column format to deplete endotoxins.

rTcTASV-C and rGST were employed as immunogens in TcTASV- or Control vaccinated groups, respectively, and also in ELISA assays and to stimulate splenocyte cultures. rTcTAV-A was employed in ELISA assays and to stimulate splenocyte cultures.

Specific anti-TcTASV-A sera were generated in mice by immunization with rTcTASV-A or in rabbits by immunization with the RQ28 peptide, which covers the NH2-term of the proteins of the TcTASV-A subfamily (García et al., 2010). This serum was used to assay TcTASV-A expression in intracellular amastigotes by immunofluorescence. Images analysis were performed using FlowJo v10.0.7 software.

### 2.3 BV::A_CAP_ baculovirus design and production

**Figure 1:**
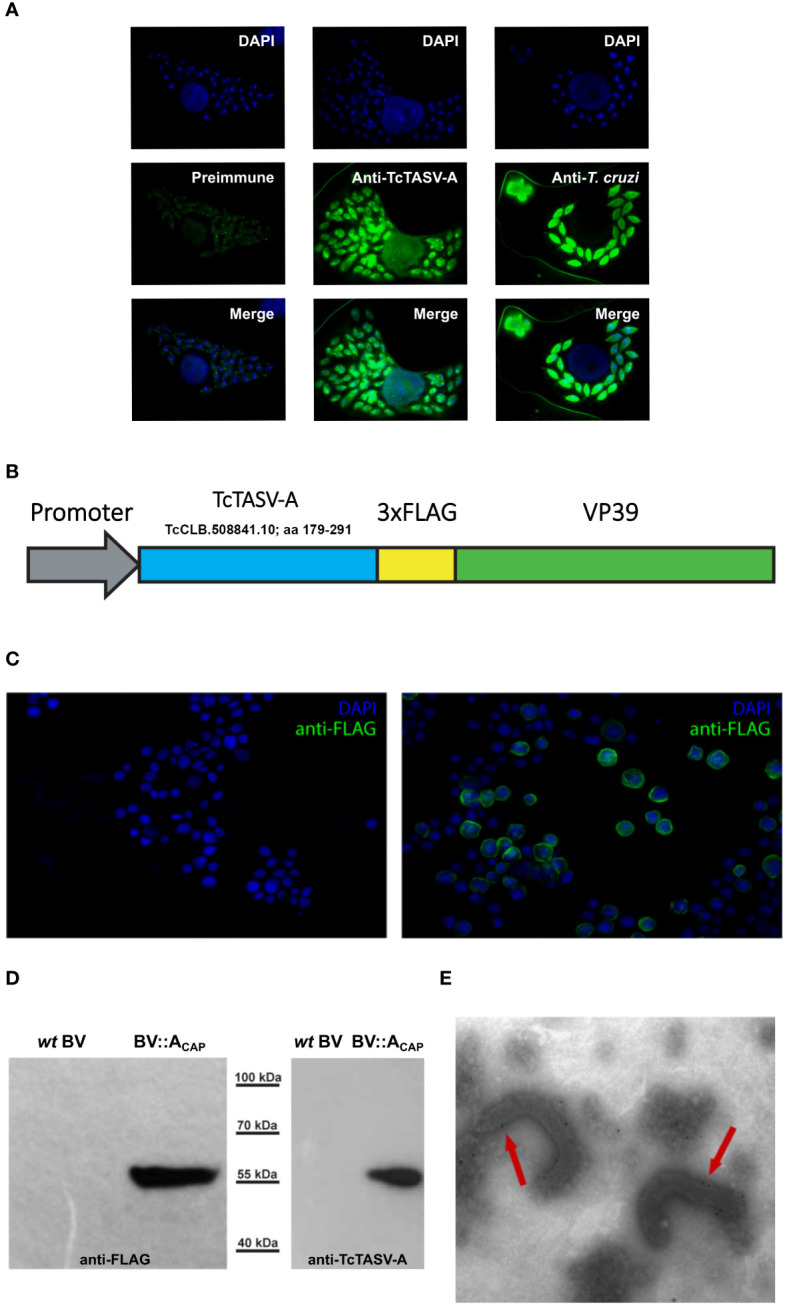
TcTASV-A: Expression in *T. cruzi* amastigotes and capsid display in recombinant baculovirus (BV::A_CAP_). **(A)** TcTASV-A is expressed in intracellular amastigotes. Vero cells were infected with *T. cruzi* trypomastigotes and the expression of TcTASV-A was assayed in amastigotes by immunofluorescence after 96 hours. Coverslips were incubated with preimmune (left), anti-TcTASV-A (center), or anti-*T. cruzi* (right) sera followed by Alexa488-labeled secondary antibody; nuclei and kinetoplastid DNA were stained with DAPI. Images were acquired with a Nikon Eclipse E600 Fluorescence Microscope. **(B–E)** Design and characterization of recombinant baculovirus displaying TcTASV-A at the capsid (BV::A_CAP_) **(B)** Schematic representation of the TcTASV-A::VP39 fusion protein (with a 3xFLAG) expressed in recombinant BV::A_CAP_. **(C)** Indirect immunofluorescence of Sf9 insect cells infected with *wild-type* (left) or recombinant BV::A_CAP_ (right) baculovirus. Anti-FLAG antibody was used, followed by anti-mouse Alexa 488. Genetic material was stained with DAPI. **(D)** Baculovirus harvested from the supernatants of Sf9 cells were assessed for the expression of TcTASV-A::VP39 fusion protein (55 kDa) in *wild-type* or recombinant BV::A_CAP_. Western blotting using anti-FLAG (left) and anti-TcTASV-A (right) antibodies. **(E)** Capsid display of recombinant TcTASV-A::VP39 was confirmed by immunoelectron microscopy (anti-FLAG antibody followed by gold-conjugated anti-mouse). No labelling was detected in *wild-type* BV (data not shown).

A synthetic fragment of a TcTASV-A gene (TcCLB.508841.10; nt 517-853, aa 179-291 (CL Brener strain) fused at the 3’ with a 3xFLAG tag, and flanked by XhoI and XbaI restriction sites, was purchased from Macrogen (TcTASV-A-3xFLAG). The TcTASV-A TcCLB.508841.10 gene selected by bioinformatic prioritization (see section 3.1) has a sequence conserved in different *T. cruzi* strains (100% identical to FN599096.1 (RA strain, TcVI), C3747_168g80 (TCC, TcVI), ECC02_003337 (Berenice, TcII), and 86% identical to TcCLB.506573.5, and other TcTASV-A genes). To construct the BV vector for capsid display of the gene of interest, TcTASV-A-3xFLAG was cloned into the XhoI/XbaI of pFBOVA_CAP_, a plasmid derived from the pFastBAC1 vector (Invitrogen) (Molinari et al., 2011). In consequence, the recombinant BV expressing the fusion construction of VP39 and TcTASV-A-3xFLAG under the polyhedrin promoter was obtained (BV::A_CAP_, **Figure 1B**). Recombinant BV::A_CAP_ was amplified and purified in *Spodoptera frugiperda* Sf9 insect cells as previously described (Molinari et al., 2011).

### 2.4 BV::A_CAP_ characterization

Expression of the fusion construct (VP39::TcTASV-A) was verified by indirect immunofluorescence assays in Sf9 cells. Briefly, Sf9 cells infected with *wild-type* BV or BV::A_CAP_ were fixed with PBS - 4% paraformaldehyde (PFA) and permeabilized with PBS containing 0.2% Triton 100 - 0,2% BSA. Labeling was carried out with a monoclonal mouse-anti-FLAG (1/1000 dilution, F1804, Sigma-Aldrich) followed by goat anti-mouse Alexa 488 (1/1000) and visualized using a Nikon Eclipse E600 microscope. The expression of the fusion construct in BV was also verified by western blotting after each passage using virions previously purified by sucrose cushion (Molinari et al., 2011). The expression of VP39::TcTASV-A at the capsid of BV was verified by immunoelectron microscopy of BV::A_CAP_ particles using an anti-FLAG antibody, as previously described (Molinari et al., 2011).

### 2.5 Immunization schemes

Six to eight weeks-old female C3H/He or BALB/c mice (4-6 per group) were immunized with two doses separated by 21 days. In the first dose 25 µg of rTcTASV-C (TcTASV-vaccinated group) or rGST (control-vaccinated group) adjuvanted with aluminum hydroxide (Sigma) were administered by s.c. route. The second dose consisted in 25 µg of recombinant proteins administered in combination with 1.10^7^ PFU of BV::A_CAP_ (TcTASV-vaccinated group) or *wild-type* BV (control-vaccinated group) without other adjuvant (i.v. under isoflurane anesthesia and i.p.). Sham-immunized mice were inoculated with equal volumes of PBS (doses 1 and 2). Serum samples were obtained by maxillary puncture five days before the first and seven days after the second immunization dose.

### 2.6 Immune response analysis

Anti-TcTASV-A, anti-TcTASV-C and anti-GST antibody production was assayed by ELISA. The plates were coated with 100 ng of recombinant proteins, blocked, and incubated with serial dilutions of murine sera. Goat anti-mouse IgG, anti-IgG1 or anti-IgG2a conjugated to peroxidase (Thermo Fisher Scientific) were used as secondary antibodies. The reaction was revealed with TMB - H_2_O_2_ in citrate buffer, stopped with sulfuric acid (1 M), and read at 450 nm using a FilterMax F5 plate reader.

Commercial arrays RayBio C-Series Mouse Cytokine Antibody Array C2 (AAM-CYT-2, RayBiotech) displaying immobilized capture antibodies specific for 32 cytokines/chemokines (6Ckine (CCL21), TACK (CCL27), Eotaxin-1 (CCL11), GCSF, GM-CSF, IL-2, IL-3, IL-4, IL-5, IL-6, IL-9, IL-10, IL-12 p40/p70, IL-12 p70, IL-13, IL-17A, IFN-gamma, KC (CXCL1), Leptin, MCP-1 (CCL2), MCP-5, MIP-1 alpha (CCL3), MIP-2, MIP-3 beta (CCL19), RANTES (CCL5), SCF, TNF RI (TNFRSF1A), TARC (CCL17), TIMP-1, TNF alpha, Thrombopoietin (TPO) and VEGF-A) were used to evaluate the cytokine secretion profile in immunized mice, following the manufacturer’s protocol. Briefly, the membranes were incubated with pooled sera (1/10 dilution) from TcTASV-vaccinated or sham-immunized mice, washed, incubated with a biotinylated-detection antibody cocktail, washed, and incubated with HRP–streptavidin. All incubations were performed overnight. Signals were detected by chemiluminescence and quantified by densitometry using the ImageJ software (NIH, Bethesda, MD, USA). Data normalization and analysis were performed according to the manufacturer’s instructions. Each array was first normalized to its internal positive controls, and then the ratio between TASV-vaccinated/sham-immunized signals was calculated for each cytokine and expressed as a log2 fold change.

To analyze cellular responses, mice were euthanized seven days after the last dose, and spleens were harvested and mechanically disrupted to obtain single-cell suspensions under sterile conditions. Cells were incubated with RBC lysis buffer (Sigma), washed, and viable cells were counted using Trypan blue 0.4% (Gibco) in a Neubauer chamber. Splenocytes (1 × 10^6^ cells/well) were co-cultured in 96 well plates in a final volume of 200 μl of RPMI-10% FBS (Natocor) with rTcTASV-C, rTcTASV-A, rGST (10 μg/ml), or without stimulus for 18 h at 37°C and 5% CO_2_. Cultures were then incubated with monensin (3 μM) for an additional 5 h, harvested, and labeled. To analyze the T cell response, cells were labeled with anti-CD8 (PE/Cy7), anti-CD4 (Alexa Fluor 647), and anti-IFNγ (PE) (all Biolegend), following the manufacturer’s instructions. All cells were fixed with PBS- 4% PFA, and samples were acquired on an LSRFortessa (BD) flow cytometer and analyzed using FlowJo software (Tree Star, Inc., Ashland, OR).

### 2.7 Functionality of antibody response

The ability of sera from vaccinated mice to mediate trypomastigote lysis by complement was assayed, as previously described (Caeiro et al., 2018). For invasion inhibition assays, 1.10^6^ CFSE-stained trypomastigotes were incubated with sera from vaccinated, infected, or control mice (dilution 1/10) for an hour at room temperature. These parasites were used to infect Vero cells on p24 plates. After 4 h of infection, the cell supernatant was replaced with fresh medium and incubated for an additional 20 h. Finally, cells were trypsinized, fixed with 0.5% PFA and the percentage of CFSE-stained cells (indicative of the presence of amastigotes) was analyzed by flow cytometry (Partec Cyflow Space) (Rodríguez et al., 2020).

### 2.8 Animal infections and parasitological follow up

Vaccinated and sham-immunized animals were challenged by i.p. route with 100 trypomastigotes of the RA strain (DTU VI). Parasitemia was evaluated 2–3 times a week, from day 9 onward by counting the number of bloodstream trypomastigotes in a hemocytometer. For counting, a 5 μl blood sample taken from the tail vein was diluted in 20 μl of RBC lysis buffer (Sigma). Parasitemia curves over time were constructed from the daily parasitemia values. The area under the parasitemia curve (AUC) was calculated individually for each animal with the corresponding tool in GraphPad Prism 6.01 software (GraphPad, San Diego, CA, USA), normalizing to the total number of days of the experiment. The AUCs of mice from the same group were averaged and means were used to make comparisons between animal groups. The survival was monitored daily.

Alternatively, surviving mice at 80 d.p.i. were immunosuppressed with cyclophosphamide (three injections of 200 mg/kg, i.p.) administered at intervals of 3 days (Calabrese et al., 1996; Pereira et al., 1996; Calabrese, 1999; Ward et al., 2022). From the 10^th^ day after immunosuppression, parasitemia and mortality were evaluated, as previously described.

### 2.9 Parasitic load analysis by qPCR

At day 75 p.i., surviving mice were euthanized and their hearts, skeletal muscles (hamstrings), and spleens were extracted for further analysis. From each tissue, 25 mg was homogenized in 500 μl of lysis buffer (EDTA 100 mM, Tris-HCL pH 7,5 10 mM, 0.5% SDS, 20 µg/ml RNAse) using a TissueRuptor II (QIAGEN). Samples were incubated for 1 h at 37°C and then for 5 h at 50°C after adding proteinase K (100 µg/ml). DNA was extracted by successive purifications in Phenol : Chloroform:Isoamyl Alcohol (25:24:1), Chloroform : Isoamyl Alcohol (24:1), and chloroform, precipitated with ammonium acetate (10 M) and ethanol, and resuspended in Tris-EDTA buffer.

Satellite DNA from *T. cruzi* was amplified with primers SATfw (GCAGTC GGCGGATCGTTTTCG) and SATrev (TTCAGTGTTGTTTGGTGTCCAGTG) and normalized to murine TNFα (TNFαfw: TCCCTCTCATCAGTTCTATGGCCCA; TNFαrev: CAGCAAGCATCTATGCACTTAGACCCC) (Duffy et al., 2009). For the qPCR reaction, 50 ng of total DNA was amplified with SATFw and SATRev primers (0.1 µM) or TNFαfw and TNFαrev (0.3 µM) and SensiFAST SYBR Lo-ROX mix (Bioline) in final volume of 10 µl. The reaction conditions were 95°C for 3 min, followed by 40 cycles of 95°C for 3 s and 60°C for 1 min. Samples were measured in triplicate using an Applied Biosystems 7500 instrument. The amplification efficacy of *T. cruzi* DNA (SAT) was normalized to that of murine TNFα (TNF) in the same tissue (SAT/TNF). Ratios of samples of the same tissue type were averaged and means were used to make comparisons between animal groups.

### 2.10 Statistical analysis

GraphPad Prism 6.01 software (GraphPad, San Diego, CA, USA) was used for statistical analyses. For ELISA assays, differences in optical density (450 nm) between vaccinated groups were determined using two-tailed unpaired Student’s *t*-test. For the cellular immune response, differences between the numbers of IFN-producing splenocytes after treatment with different stimuli were analyzed by two-way ANOVA plus Bonferroni’s post-test. For lytic antibody differences, inhibition of invasion analysis, and parasitic load by qPCR, comparisons were made using one-way analysis of variance (ANOVA), adding Tukey’s post-test when necessary. Differences in parasitemia levels were determined using the Mann–Whitney U test. For survival rate, differences between curves were analyzed by log-rank test (Mantel-Cox test).

## 3 Results

### 3.1 Selection of a novel vaccine antigen of the TcTASV-A subfamily: expression in amastigotes and at the capsid of recombinant baculovirus

To prioritize the selection of *T. cruzi* genes that (1) were overexpressed at higher levels in mammals with respect to insect stages (mRNA), (2) have been identified by proteomics, without orthologs in other organisms (from mammals to trypanosomatids), and (3) have predictions for transmembrane domain/s, a search engine was carried out (TriTrypDB; Amos et al., 2022) (https://tritrypdb.org/tritrypdb/app/workspace/strategies/import/13776b69c14ec577). This search retrieved one gene, TcCLB.508841.10, which belonged to the TcTASV-A subfamily. Peptides from several TcTASV-A proteins have also been detected in proteomics of trypomastigotes, extracellular vesicles and amastigotes (Atwood et al., 2005; Brunoro et al., 2015; Mandacaru et al., 2019). In addition, we have previously reported the expression of TcTASV-A subfamily in trypomastigotes: (García et al., 2010), its secretion associated to EVs (Caeiro et al, 2018), and the utility of TcTASV-A in diagnostic tests (Floridia-Yapur et al., 2016, 2019). Although the serology tests included a protein of each TcTASV-A and TcTASV-C subfamilies, TcTASV-A was more sensitive than TcTASV-C in detecting human infections in young adults, children and dogs without evidence of circulating parasites (Floridia-Yapur et al., 2016, 2019), suggesting its expression during the chronic infection (i.e. amastigotes). The expression of TcTASV-A in intracellular amastigotes was confirmed using immunofluorescence (**Figure 1A**). As TcTASV-A is one of the two major TcTASV subfamilies and is conserved in all *T. cruzi* lineages (García et al., 2010; Bernabó et al., 2013), we decided to add TcTASV-A TcCLB.508841.10 as an additional vaccine antigen to the already assayed and promising rTcTASV-C (Caeiro et al., 2018, 2020).

An alternative TcTASV-A presentation platform employing recombinant baculovirus (BV) was designed. A fragment of TcTASV-A TcCLB.508841.10 was fused to a second copy of the main BV capsid protein (VP39), downstream of a strong expression promoter (**Figure 1B**). Heterologous TcTASV-A expression was verified in insect cells (**Figure 1C**), in cell-derived purified viruses (**Figure 1D**) and at the BV capsid, as confirmed by electron microscopy (**Figure 1E**). Recombinant BV expressing TcTASV-A at the capsid (BV::A_CAP_) was used in the immunization assays.

### 3.2 TcTASV vaccination elicits robust immune responses

**Figure 2:**
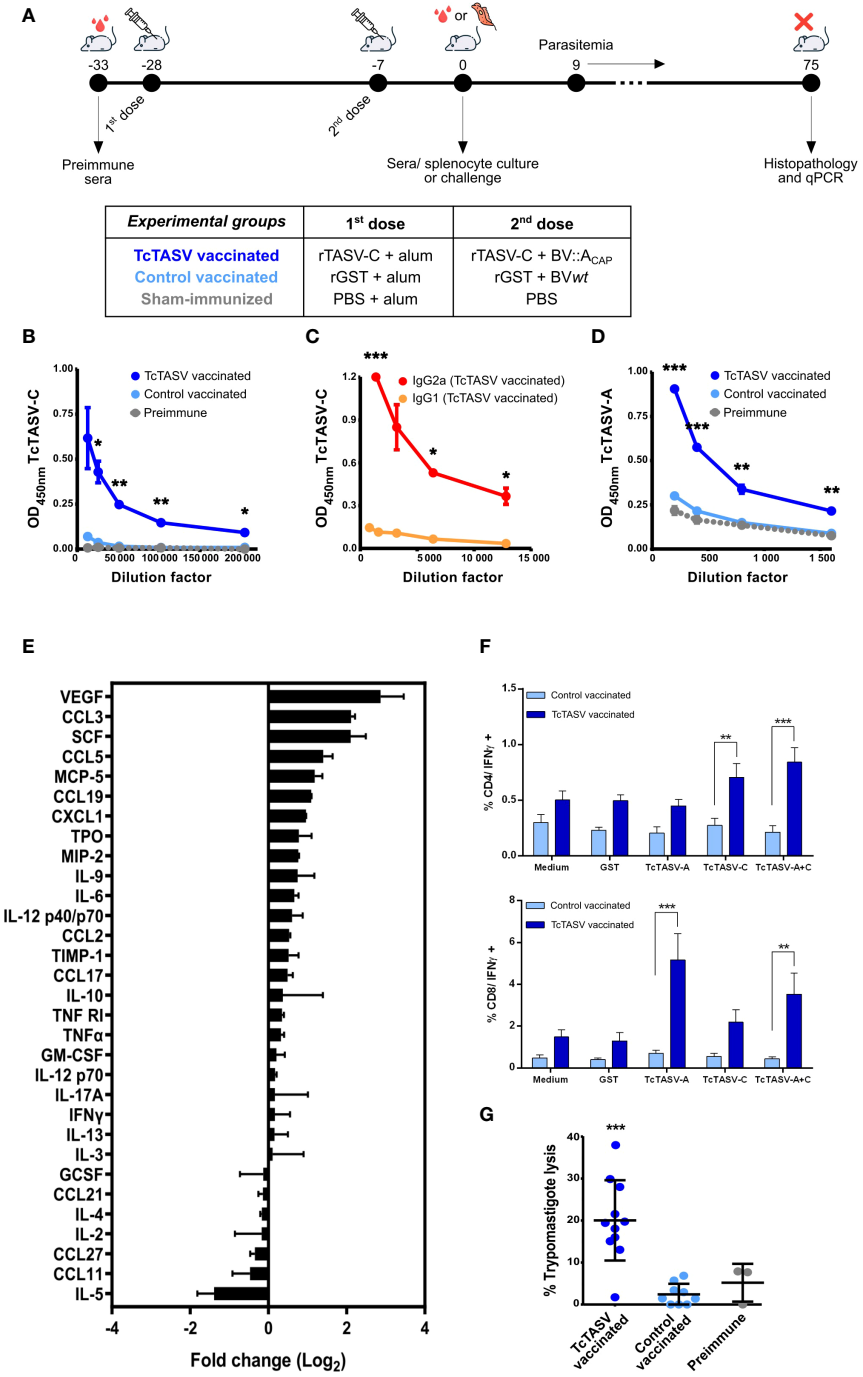
Vaccination with TcTASV and baculovirus elicits robust immune responses. **(A)** Immunization scheme and experimental design. Animals were immunized with recombinant proteins (rTcTASV-C or rGST) in the first dose and with recombinant proteins plus BV in the second (rTcTASV-C+BV::A_CAP_ or rGST+*wt*BV); sham-immunized animals were injected with PBS. Immunological and parasitological parameters (after challenge) were evaluated. **(B-D)** Evaluation of antibody responses by ELISA in immunized animals. **(B)** Total IgG, **(C)** IgG1 and IgG2a isotype profile against TcTASV-C, and **(D)** total IgG against TcTASV-A were evaluated in sera obtained seven days after the last immunization dose. The OD values obtained for GST were subtracted and the anti-TcTASV specific response was plotted. Two-tailed unpaired Student’s t-test between TcTASV- and control-vaccinated groups (*p ≤ 0.05; ** p≤ 0.005; ***p ≤ 0.0005). Mouse strain: C3H/He; N= 5/group. Similar results were observed in 3 independent experiments. **(E)** Cytokine and chemokine profiles in vaccinated mice. Commercial arrays displaying immobilized capture antibodies specific for 32 cytokines were used to evaluate the cytokine secretion profile in sera of immunized mice at 7 days after last dose. The membranes were incubated with pooled sera from TcTASV-C-vaccinated or sham-immunized mice, developed by chemiluminescence and quantified by densitometry using the ImageJ software. Each array was first normalized to its internal positive controls, and then the ratio between TcTASV-vaccinated/sham-immunized signals was calculated for each cytokine and expressed as a log2 fold change. **(F)** Cellular immune responses. Immunized mice were euthanized seven days after the second dose, and splenocytes were cultured with media alone or stimulated with rGST, rTcTASV-C, rTcTASV-A, or both rTcTASV-C and rTcTASV-A (10 μg/mL) for 24 hours. Cells were harvested and surface labeled for CD4, CD8, and intracytoplasmic IFNγ. Fluorescence acquisition and quantification was performed using flow cytometry. Upper panel: IFNγ production by CD4+ cells. Lower panel: IFNγ production by CD8+ cells. Two-way ANOVA plus Bonferroni’s post-test (**p ≤ 0.005; ***p ≤ 0.0005). **(G)** Complement-mediated lysis of trypomastigotes by sera from TcTASV-vaccinated mice. Sera from TcTASV-, control- vaccinated mice or preimmune sera were decomplemented and incubated with cell-derived trypomastigotes (RA strain) in the presence (or not) of fresh human serum as an external source of complement at 37°C. After 1 h, live parasites were quantified by microscopy. The lysis percentage was calculated as the ratio of live parasites incubated with and without complement (established as 100% survival). One-way ANOVA plus Tukey’s post-test (***p ≤ 0.0005; differences calculated vs. pre-immune sera).

Mice were immunized with recombinant proteins (rTcTASV-C or rGST, 1^st^ dose) and boosted 21 days later with rTcTASV-C and BV::A_CAP_ (or rGST and *wt*BV) as described in section 2.5 and shown in **Figure 2A**. Sham-immunized mice were inoculated with PBS in both doses. BV-based vaccine biosafety was assessed in tissue sections of immunized animals; no histological alterations were observed (**Supplementary Figure 1**).

Specific anti-TcTASV-C IgG antibody responses (**Figure 2B**), with titers higher than 200,000, and mainly of subclass IgG2a (**Figure 2C**), which indicated a main Th1 response, were detected. An appropriate anti-TcTASV-A humoral response was also elicited, although the titer was lower (>1,600) than that obtained for TcTASV-C (**Figure 2D**).

Elevated serum levels (twice or more than controls) of VEGF, a proangiogenic cytokine, and chemokines CCL3, CCL5, and CCL19, which are chemoattractant cytokines that promote the migration of monocytes, macrophages, DCs, and other innate response cells were detected in TcTASV vaccinated mice (**Figure 2E**). Populations of CD4+ T lymphocytes producing IFNγ after re-stimulating cultures with rTcTASV-C or rTcTASV-A+C were identified in the splenocytes isolated from TcTASV-vaccinated mice (**Figure 2F**). Similarly, populations of CD8+ T lymphocytes producing IFNγ were identified after rTcTASV-A or rTcTASV-A+C stimulation (**Figure 2F**). In both cases, these populations were significantly higher than those observed in control-vaccinated mice (rGST + BV*wt*) stimulated with the same antigens. No differences between the groups were observed after stimulation with any other antigen. Thus, this vaccination scheme appeared to generate CD8+ T lymphocytes responsive to TcTASV-A and CD4+ T lymphocytes responsive to TcTASV-C.

Antibodies present in sera from TcTASV-vaccinated mice, but not in control vaccinated mice, were functional and able to lyse culture-derived trypomastigotes in the presence of an external complement source (**Figure 2G**). However, the cellular invasion of Vero cells was not affected when trypomastigotes were previously incubated with sera from TcTASV- or control-vaccinated mice (**Supplementary Figure 2**).

### 3.3 TcTASV vaccinated mice survive a lethal challenge with *T. cruzi* and present reduced parasitic load in chronic infection

**Figure 3:**
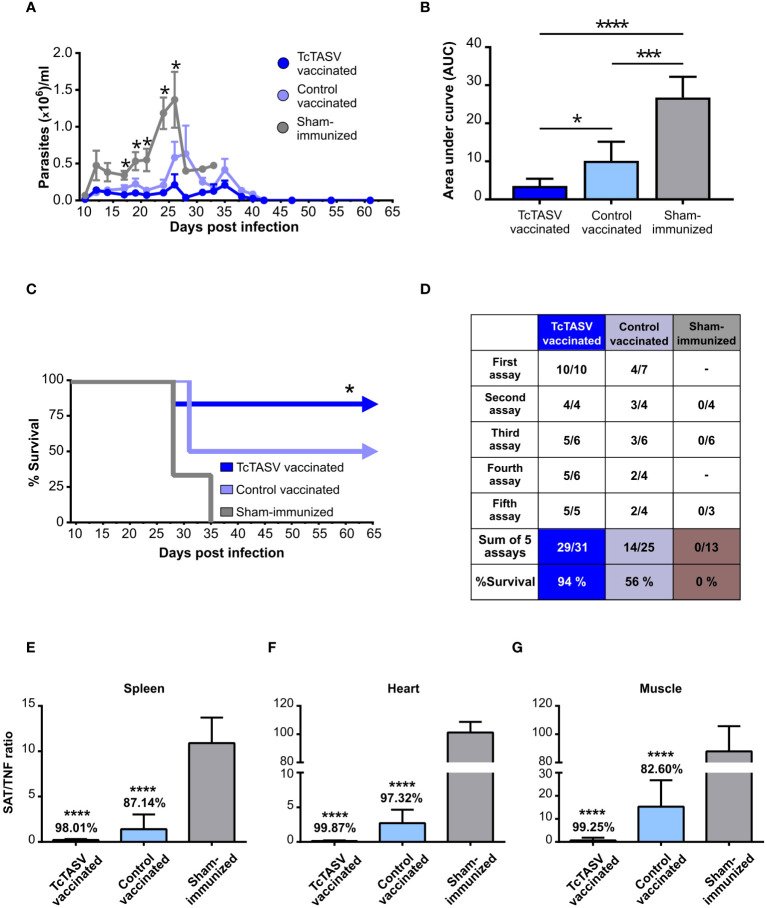
Immunization with rTcTASV-C and BV::A_CAP_ protects mice from challenge with a highly virulent strain. Immunized C3H/He mice were challenged with a lethal inoculum of *T. cruzi* (100 tryp, i.p., RA strain DTU TcVI) 7 days after the last immunization dose. Acute infection **(A–D)**. **(A)** Parasitemia was evaluated from day 9 post-infection (Mann-Whitney test, *p ≤ 0.05); **(B)** Area under the curve (AUC) of parasitemia in relative units, normalized to total days of infection (One-way ANOVA + Tukey’s post-test, *p ≤ 0.05; ***p ≤ 0.0005; ****p≤ 0.0001); **(C)** Survival rates (third assay; log rank test; *p ≤ 0.05). **(D)** Summary of survival parameters for different immunization and challenge assays in C3H/He mice. Chronic infection **(E–G).** Immunized and challenged C3H/He mice that were alive at chronic infection were euthanized at 75 d.p.i; otherwise, tissues were removed from mice at the time of death. Parasitic load was determined by qPCR, using the ratio of amplification between SAT (*T. cruzi* satellite DNA) and TNFα (murine gene, unique copy). **(E)** Spleen. **(F)** Heart. **(G)** Isquiotibial muscle. The number above each bar indicates the percentage reduction of each group with respect to the control group injected with PBS, calculated as the quotient between the means of both groups. Ordinary one-way ANOVA plus Tukey’s post-test (****p ≤ 0.0001).

TcTASV-vaccinated, control-vaccinated, and sham-immunized mice were challenged with the highly virulent RA strain (DTU TcVI); in the absence of any protection, this dose induced 100% mortality at 25-35 days post infection. TcTASV-vaccinated animals showed significantly lower levels of parasitemia (**Figure 3A**) and a 9 times reduction in the number of circulating parasites in the acute phase compared to that in sham-immunized mice (AUC, **Figure 3B**). Four independent immunization and challenge assays were performed using C3H/He mice. TcTASV-vaccinated animals had an overall survival rate of 94% (29/31, ranging from 83 to 100% survival in different assays), whereas all control sham-immunized mice died before day 35^th^ post infection (**Figures 3C, D**). Of note, an average of 56% of the control vaccinated animals survived at the end of the experiment (14/25). To determine whether our immunization schedule affected chronic infection, tissues were obtained from infected mice the day after their deceased, or when the experiment was concluded in surviving mice (75 d.p.i.), and the presence of *T. cruzi* was analyzed by qPCR. A reduction of more than 98% in parasite load in spleens, hearts, and skeletal muscles was observed, compared with the same tissues obtained from sham-immunized mice inoculated with PBS (**Figures 3E–G**). In addition, inflammatory infiltration, parasitic foci, and histological damage in the heart and skeletal muscle of TcTASV-vaccinated animals showed better scores than control animals (either control-vaccinated or sham-immunized mice) (**Supplementary Table 1**).

### 3.4 TcTASV vaccination allows to control the infection after reactivation by immunosuppression and after rechallenge with *T. cruzi*

**Figure 4:**
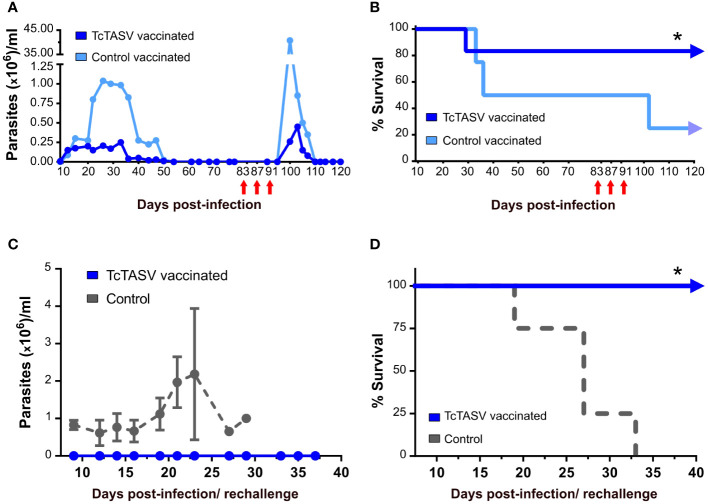
TcTASV-vaccinated and infected mice in the chronic phase control the reactivation of parasitemia and a second challenge with *T. cruzi*. **(A, B)** Mice were immunized and challenged as previously described. TcTASV- (n= 4) and control-vaccinated (n= 4) chronically infected mice with no detectable bloodstream parasites were immunosuppressed with three doses of cyclophosphamide (red arrows). **(A)** Parasitemia and **(B)** survival rates (log rank test; *p ≤ 0.05). **(C, D)** TcTASV-vaccinated and chronically infected mice (n= 4) were re-challenged with 10,000 tryp/mouse (100X lethal dose). The control animals (n= 4) were naïve mice. **(C)** Parasitemia and **(D)** survival rates (logrank test; *p ≤ 0.05). Mouse strain:C3H/He.

To evaluate the chronic presence of *T. cruzi* in tissues after the initial control of parasitemia and, indirectly, the immunological memory response, surviving mice were immunosuppressed by cyclophosphamide treatment. TcTASV-vaccinated mice showed a marked but brief increase in parasitemia (much lower than that observed in control vaccinated mice) followed by a fast control of circulating parasites, which were no longer detected at 110 d.pi (i.e., ~30 days after immunosuppression) (**Figure 4A**). In addition, 100% of the TcTASV-vaccinated, infected, and immunosuppressed mice survived this treatment, controlling the parasitemia reactivation, while the percentage was 50% for control-vaccinated mice (**Figure 4B**). In another assay, TcTASV-vaccinated animals that had survived *T. cruzi* infection and achieved the chronic phase were re-challenged 170 days post infection with a 100X lethal dose of trypomastigotes. As a control, a group of age-matched and sex-matched unvaccinated animals was also infected. While all control animals developed parasitemia and died before 35 d.p.i., no parasites or mortality were detected in the TcTASV-vaccinated animals (**Figures 4C, D**).

### 3.5 TcTASV vaccination is effective and protects against *T. cruzi* infection in another mouse strain

**Figure 5:**
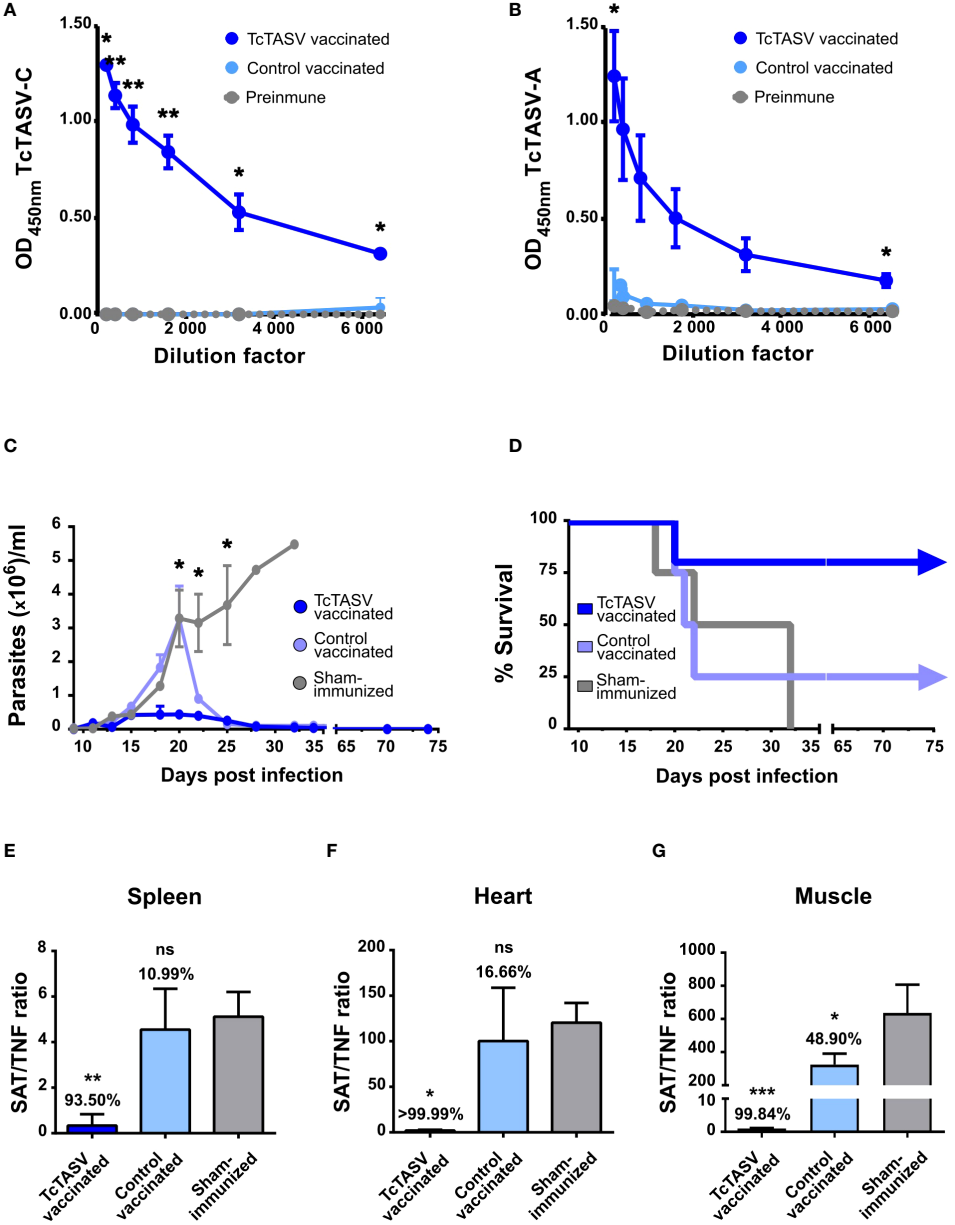
TcTASV vaccination also worked efficiently in a more susceptible mouse strain. Balb/c mice were immunized as described for the C3H/He mice. Antibody reactivity against **(A)** TcTASV-C and **(B)** TcTASV-A was determined by ELISA, in sera obtained before the first and after the second dose of immunization. Two-tailed unpaired Student’s t-test between TcTASV- and control-vaccinated groups (*p ≤ 0.05; **p ≤ 0.005). **(C–G)** Alternatively, mice (n = 4/group) were challenged with 100 trypomastigotes (RA strain, TcVI). **(C)** Parasitemia (Mann-Whitney test, *p ≤ 0.05) and **(D)** survival were monitored up to day 75, when the surviving mice were euthanized. Parasitic tissue load was analyzed in the spleen **(E)**, heart **(F)**, and isquiotibial muscle **(G)** by qPCR. The number above each bar indicates the reduction percentage of each group against the sham-immunized mice, calculated as the quotient between the means of both groups. Ordinary one-way ANOVA plus Tukey’s post-test (*p ≤ 0.05; **p ≤ 0.005; ***p ≤ 0.0005); ns, non-significant difference).

Given the results obtained for C3H/He mice, the same immunization scheme was used for BALB/c mice. Specific anti-TcTASV-C (**Figure 5A**) and anti-TcTASV-A (**Figure 5B**) antibody responses, both with titers higher than 6,400 were detected. Two independent immunization and challenge assays were performed using BALB/c mice. TcTASV-vaccinated mice challenged with *T. cruzi* (TcVI, RA strain) showed the lowest levels of parasitemia (**Figure 5C**). TcTASV-vaccinated animals had an overall survival rate of 90% (9/10, ranging from 80 to 100%), while control-vaccinated and sham-immunized animals showed a survival rate of 38% (ranging from 25% to 50%) and 0% survival, respectively (**Figure 5D**). Significant reductions in parasite tissue load in chronically infected mice were observed in the spleens (**Figure 5E**), hearts (**Figure 5F**), and skeletal muscles (**Figure 5G**) in TcTASV-vaccinated animals.

Overall, the results presented here demonstrate that TcTASV vaccination triggers an adequate immune response that allows control of both acute and chronic *T. cruzi* infection. The strength of this vaccination platform is supported by its efficacy against a highly virulent *T. cruzi* strain in mice with distinct genetic backgrounds.

## 4 Discussion

A vaccine against *T. cruzi* is desirable not only as a control strategy against Chagas’ disease, but also considering its beneficial economic impact because of the elevated global burden of healthcare costs due to disease complications (Hotez et al., 2014; Bartsch et al., 2018). Several vaccines against *T. cruzi* have been pre-clinically tested in animal models, using different antigens and platforms. Most assayed antigens are well-characterized parasite virulence factors belonging to large multigene families, such as *trans*-sialidase, ASP-2, and cruzipain (Tzelepis et al., 2008; Haolla et al., 2009; Bontempi et al., 2015, 2017; Cerny et al., 2020; Moraschi et al., 2021; Castro et al., 2023; Jha et al., 2023). However, to date, none antigen-defined vaccines have been able to induce an immune response that avoids an initial infection with the parasite. Considering the antigenic complexity of *T. cruzi* and its life cycle, an effective vaccine should be multi-antigenic and should combine several key parasite molecules. If infection cannot be avoided, a prophylactic vaccine should induce a substantial decrease in parasite burden in tissues, which will eventually diminish morbidity and mortality rates. In recent years, special attention has been paid to the use of glycoantigens, chimeric antigens, novel adjuvants, and live platforms (Nogueira et al., 2013; Sanchez Alberti et al., 2017; Portillo et al., 2019; Bivona et al., 2020; Lokugamage et al., 2021; Rodrigues da Cunha et al., 2021; Cai et al., 2022; Pacini et al., 2022). Recently, Jha et al. (2023) demonstrated that a parasite attenuated by the deletion of cyclophilin-19 completely prevents *T. cruzi* infection. Although this study showed that sterilizing immunity against *T. cruzi* can be achieved by immunization, the use of live parasites as vaccines still requires additional work to evaluate its safety. Other live vaccine platforms, such as viral, adenoviral, or bacterial vectors, can present potential limitations due to pre-existing anti-vector immunity caused by the overlapping of endemic areas between *T. cruzi* and viral vectors (i.e., yellow fever) or by previous vaccination regimens that could hinder their efficacy. Concerning baculovirus, its most well-known use is as an expression system for producing recombinant proteins applicable to vaccines, such as in the case of the HPV vaccine (Gardasil^®^). The application of BV as vectorized vaccines, however, is currently limited to pre-clinical trials in animal models. Nevertheless, the inherent advantages of baculovirus as vectorized vaccine platforms are noteworthy. The simplicity and low cost of methods for obtaining recombinant baculoviruses, coupled with their non-replicative nature in mammalian cells and their ability to tolerate the insertion of extensive heterologous gene sequences, are key aspects. Furthermore, the expression of heterologous proteins in the baculovirus capsid or surface allows for the induction of robust cellular or humoral responses, respectively (Molinari et al., 2011; Tavarone et al., 2017). Finally, unlike other vectorized vaccines, baculovirus enables the transport of pathogen components within its own structure, eliminating the need for antigen expression in the host. This feature ensures the immediate availability of the antigen, prompting a direct and rapid activation of the immune system.

In this study, we demonstrated the effectiveness of a novel immunization strategy that employs *T. cruzi* antigens of the conserved and unique TcTASV family delivered in a combined formulation as recombinant proteins and displayed at the capsid of recombinant baculovirus (BV). The use of BV as a vehicle for vaccine delivery is a novel approach that would rule out the concern of pre-existing anti-vector immunity, because they are not able to infect mammals. The vaccination scheme presented in this study affected both early (acute, i.e., parasitemia) and long (chronic, i.e., tissue parasite burden) infection, and significantly improved the survival of animals. Moreover, vaccination induces an immune response that allows animals to control reactivation of the infection after immunosuppression.

Several years ago, in the search for novel antigens for vaccine development, our group discovered the *T. cruzi* TcTASV family, which interestingly has no orthologs in other species, is highly conserved among the heterogeneous *T. cruzi* lineages and is mainly (but not solely) expressed in trypomastigotes and secreted into extracellular vesicles (García et al., 2010; Bernabó et al., 2013; Caeiro et al., 2018). The TcTASV-A and TcTASV-C subfamilies are the most numerous and best-characterized members of the TcTASV family. In particular, TcTASV-C is a parasite virulence factor involved in the establishment of the initial infection and is highly expressed in bloodstream trypomastigotes *in vivo* (Bernabó et al., 2013; Caeiro et al., 2018). In addition, a TcTASV-C gene was identified in a pool of protective antigens selected using an expression library immunization approach employing a trypomastigote-enriched cDNA library (Tekiel et al., 2009). An effective vaccine against Chagas’ disease should adequately combine antigens, adjuvants, and delivery systems that trigger concerted and appropriate innate, humoral, and cell-mediated immunity (Acosta Rodríguez et al., 2019; Rios et al., 2019; Bivona et al., 2020; Ferragut et al., 2021; Pérez‐Mazliah et al., 2021; Magalhães et al., 2022). In addition, it is desirable for a prophylactic vaccine to eradicate as many circulating parasites in the bloodstream as possible, thus disrupting the initial phase of *T. cruzi* infection. This also reduces the number of parasites available to reach target organs and establish the intracellular infection. In previous studies, we carried out different schemes of immunization with the TcTASV-C subfamily, that is, as prime (DNA) and boost (protein) (Caeiro et al., 2018) or with recombinant proteins and different combinations of adjuvants (Caeiro et al., 2020). Overall, these trials systematically delayed the appearance of bloodstream trypomastigotes, stimulated strong antibody-mediated responses, and increased the survival of mice by up to 40% after challenge with a highly virulent strain. We hypothesized that the addition of an antigen expressed in amastigotes and other delivery platforms and/or adjuvants would improve the protective ability of the rTcTASV-C-based vaccine. A member of the TcTASV-A subfamily (TcCLB.508841.10) was selected as an additional vaccine candidate and delivered into a recombinant budded baculovirus (BV). The construct structure of BV::A_CAP_ (its fusion to VP39), the immunofluorescence, western and electron microscopy images, together with our previous experience working with BVs, strongly indicates that TcTASV-A-3xFLAG-VP39 is properly displayed at the BV capsid. Immunization with rTcTASV-C in the first dose followed by a boost with rTcTASV-C and BV::A_CAP_ elicited innate, humoral, and cellular immune responses that clearly improved the infection outcome of vaccinated mice by significantly diminishing mortality and tissue parasitic load, with more than 90% of mice surviving after challenge with a highly virulent *T. cruzi* strain.

The initial desirable response against *T. cruzi* is mediated by IFNγ and includes the recruitment of macrophages, natural killer cells, and DCs, among others. Indeed, after TcTASV vaccination, mice presented a chemokine profile in peripheral circulation that supported the recruitment of these innate immune cells. Our vaccination scheme probably activates the innate immune response in a manner that further favors infection control in immunized animals. This basal activation could allow the host to mount an effective response and surpass nonspecific polyclonal activation and other immune evasion mechanisms induced by *T. cruzi* in the early phase of infection (Reina-San-Martín et al., 2000; Hervas-Stubbs et al., 2007; Bryan et al., 2010; Bermejo et al., 2011). Vaccinated animals show a potent antibody response, with higher titers than those previously obtained in immunizations with rTcTASV-C adjuvanted with aluminum hydroxide, saponin, and/or U-Omp19 (Caeiro et al., 2018, 2020). In addition, functional antibodies triggered by vaccination contribute to the complement-mediated lysis of trypomastigotes, which is a desirable immune mechanism that interferes with the initial phase of infection (Lidani et al., 2019; Ramírez-Toloza et al., 2021). The improvement in humoral response may be due to the already reported adjuvant capacity of the baculovirus (Abe and Matsuura, 2010; Molina et al., 2021). Additionally, the major isotype in the antibody population was IgG2a, indicating a predominantly Th1 profile, which is the most appropriate for controlling *T. cruzi* (Kumar and Tarleton, 2001). Vaccination also stimulated TcTASV-A-specific IFNγ-secreting CD8+ T cell populations, which could act directly on infected cells. A TcTASV-C-specific cellular CD4+/IFNγ+ response that may be involved in the activation of plasma cells secreting anti-TcTASV-C antibodies, as discussed above, was also induced by vaccination. The reflection of an adequate immune response driven by our vaccination protocol was evidenced by the effective control of *T. cruzi* in both acute and chronic phases. On one hand, overall parasitemia (i.e. bloodstream trypomastigotes) was lower in immunized animals than in control animals. On the other hand, the parasitic load monitored to quantify chronic infection showed a notable (always higher than 93,5%) decrease in the relative level of parasites in the heart, skeletal muscle, and spleen. The results were similar for the different mouse strains, which strengthened the soundness of this vaccination protocol and the antigens used. In addition, the safety of BV as a delivery platform was demonstrated by tissue histopathology in immunized animals.

Recombinant baculovirus have never been explored as a vaccine platform for *T. cruzi*, but there are previous reports on its use to deliver antigens for other infectious diseases. Immunization with recombinant baculovirus displaying the rhoptry protein ROP4 protects mice from lethal challenge with *T. gondii* by inducing mucosal, cellular, and humoral immunity (Yoon et al., 2021). The ability of BV-based vaccines to induce strong immune responses and protection by displaying heterologous antigens has also been demonstrated for *Plasmodium* (Yoshida et al., 2018) and several viruses, including a recent work on SARS-CoV2 (Abe et al., 2003; Premanand et al., 2018; Wei et al., 2021; Xue et al., 2022). However, all of these studies have been carried out displaying heterologous antigens on the BV surface. In contrast, in the present study, we attempted to potentiate the cellular response by expressing a TcTASV-A protein at the BV capsid, which is one of the novelties of our study. It has been reported that the quality and intensity of the immune response triggered by BV vaccination depend on antigen localization. Our previous studies demonstrated that immunization with BV displaying the model antigen OVA at the capsid was much more appropriate than surface display to stimulate antigen-specific T CD8+ responses with IFN production (Molinari et al., 2011, 2018; Tavarone et al., 2017). The experimental settings of Molinari et al. (2011) were adopted and established as the initial conditions assayed in this study (i.e., BV inoculum, route of administration, and time after the last dose to measure immune responses and challenge) and prompted cellular and humoral immune responses that affected the control of the infection.

Surprisingly, control-vaccinated mice immunized with the fusion-tag GST protein and *wild-type* BV showed intermediate levels of protection for all evaluated parameters (parasitic load, parasitemia, and survival). Despite being non-specific to *T. cruzi*, this protection was truly induced by BV, because all animals in the sham-immunized group (inoculated with PBS) died before 35 d.p.i., suggesting that the immune response triggered by BV is beneficial for partial protection against *T. cruzi*. Knowing that *wild-type* BV can induce innate immune responses through the production of IFN-α and IFN-γ, partial protection in our vaccination protocol by *wild-type* BV could be mediated by the stimulation of a virus-like response. In this sense, it was recently reported that a virus-like infection response is the most upregulated pathway *in vitro* after the interaction between dendritic cells and *T. cruzi* (Gil-Jaramillo et al., 2021). Our results also agree with those reported by Emran et al., who showed that immunization with BV in the absence of parasitic antigens protected against *Plasmodium* infection (Emran et al., 2018). In line with this, it was reported that BV triggered an antigen-nonspecific response, detected 6 h after inoculation and maintained for up to 7 days, which completely killed the liver stage of the parasite (Iyori et al., 2021). Similarly, intranasal administration of *wild-type* BV conferred sufficient protection to survive the lethal challenge of influenza 21 days after immunization (Abe et al., 2003). A possible explanation for these observations could be that BV immunization stimulates innate and trained innate immune responses, which are mainly mediated by NK cells, macrophages, and monocytes (Netea et al., 2011; Netea and Joosten, 2016). Increasing evidence from massive population vaccinations has shown that live vaccines can induce cross-protection against unrelated diseases (Benn et al., 2013). These beneficial non-specific effects have been well-documented for BCG and, more recently, for adenoviral vectors used as vaccine delivery systems for COVID-19 (Jeyanathan et al., 2022; Murphy et al., 2023). Although not analyzed here, we postulate that an additional benefit of BV-based vaccination could be the triggering of a (trained) immune response that complements the antigen-specific anti-*T. cruzi* response. Therefore, the protection observed in this work in the TcTASV-vaccinated group is the result of the combination of antigens, adjuvants, and the delivery system employed.

Another strength of our vaccination scheme is its ability to prevent disease reactivation after immunosuppression in the chronic phase, and after a repeated exposure to *T. cruzi*. Considering that the ability of a cured mouse, or effectively protected by vaccination in this case, to resist re-challenges is dependent on the size of the inoculum, vaccinated mice that survived the initial infection were re-challenged with a 100X lethal dose of parasites. TcTASV-vaccinated animals completely avoided the appearance of bloodstream trypomastigotes, which indicated that the effector and memory immune responses mounted in TcTASV-vaccinated animals after the first challenge were fully effective. Although we are aware that an additional group of chronically infected unvaccinated mice would have been the most suitable control group, this was not possible because none of the infected unvaccinated mice survived beyond 35 d.p.i. Data reported by other groups using less virulent strains demonstrated that, while some immunization protocols protected mice from rechallenge, *T. cruzi* infection alone did not (Bustamante and Tarleton, 2015; Chowdhury et al., 2020; Mann et al., 2020; Ward et al., 2022). The effectiveness of our immunization protocol in protecting against re-challenges is highly relevant because, in the context of natural human infections, re-infections are frequent and associated with the risk of chronic manifestations of the disease (Monje-Rumi et al., 2015; Tomasini et al., 2017; Prescilla-Ledezma et al., 2021; Olivo Freites et al., 2022; Hakim et al., 2023). Complementary, after immunosuppression, TcTASV-vaccinated and infected mice quickly recovered from the reactivation of the infection, controlling parasitemia to undetectable levels without mortality, while mortality and extremely high levels of parasitemia were detected in control-vaccinated and infected mice. Again, these results indicate that there was a differential immune profile associated with TcTASV-vaccination that allowed infected mice to control reactivation of the infection.

In conclusion, we demonstrated that immunization with TcTASV along with recombinant baculovirus (which works both as an adjuvant and delivery platform) is a promising vaccine formulation against *T. cruzi*. Our immunization strategy interferes with both the acute and chronic phases of *T. cruzi* infection, triggering an immune response that diminishes parasitemia and parasite load in tissues and increases the survival of mice. In addition, the reactivation of infection after immunosuppression was accurately controlled by the anti-TcTASV response. It should be stressed that our vaccination protocol was effective in an extremely challenging model, considering that we employed a highly virulent strain that causes 100% mortality in the absence of any protection. However, we acknowledge that these promising results should be broadened by, for example, evaluating its performance with other *T. cruzi* strains, and delaying the time of infection. The impact of this vaccination scheme also highlights the relevance of the TcTASV family in the biology of parasite infection and supports further studies by combining it with other *T. cruzi* antigens to design a multiantigenic prophylactic vaccine. We believe that the present results employing a combination of a baculovirus platform and the TcTASV family are promising for the development of a protective vaccine against Chagas disease.

## Data availability statement

The original contributions presented in the study are included in the article/**Supplementary Material**. Further inquiries can be directed to the corresponding author.

## Ethics statement

The animal study was approved by Comité Institucional para el Cuidado y Uso de Animales de Experimentación de la Universidad Nacional de San Martín (CICUAE-UNSAM); https://www.unsam.edu.ar/investigacion/cicuae.asp. The study was conducted in accordance with the local legislation and institutional requirements.

## Author contributions

YM: Conceptualization, Data curation, Formal analysis, Investigation, Methodology, Writing – original draft, Writing – review & editing. LC: Formal analysis, Investigation, Methodology, Writing – review & editing. MC: Investigation, Methodology, Writing – review & editing. MP: Formal analysis, Investigation, Methodology, Writing – review & editing. GM: Investigation, Methodology, Writing – review & editing. OT: Conceptualization, Formal analysis, Methodology, Resources, Writing – review & editing. MPM: Conceptualization, Formal analysis, Investigation, Methodology, Resources, Writing – review & editing. VT: Conceptualization, Data curation, Formal analysis, Funding acquisition, Investigation, Methodology, Project administration, Resources, Supervision, Visualization, Writing – original draft, Writing – review & editing.

## Supplementary material

Supplementary Figure 1 Safety of the immunization protocol: Vaccination with rTcTASV-C and BV::A_CAP_ does not affect the tissular architecture. Heart and skeletal muscles of TASV- and control-vaccinated, and sham-immunized mice were obtained 7 days after the last dose. Tissue sections were stained by H&E and analyzed under light microscopy. Magnification 100X.

Supplementary Figure 2 Sera from TcTASV-vaccinated mice do not affect cellular invasion. CFSE-labeled trypomastigotes were incubated with sera from vaccinated, control or infected mice for 60 minutes and then used to infect Vero cells, as indicated in Section 2.7. The percentage of infected cells was determined at 24 h by flow cytometry. Ordinary one-way ANOVA plus Tukey’s post-test (group a vs group b: p ≤ 0.05).

Supplementary Table 1 Scoring of tissue inflammation and parasite foci by histopathology on H&E-stained sections of the heart and skeletal muscle from TcTASV vaccinated, control vaccinated and sham-immunized mice.
