# Supplementary figures and images for: Vaccination with parasite-specific TcTASV proteins combined with recombinant baculovirus as a delivery platform protects against acute and chronic Trypanosoma cruzi infection
Source: Front Cell Infect Microbiol. 2024 Feb 28;14:1297321. doi: 10.3389/fcimb.2024.1297321 (PMC10933115; doi:10.3389/fcimb.2024.1297321)

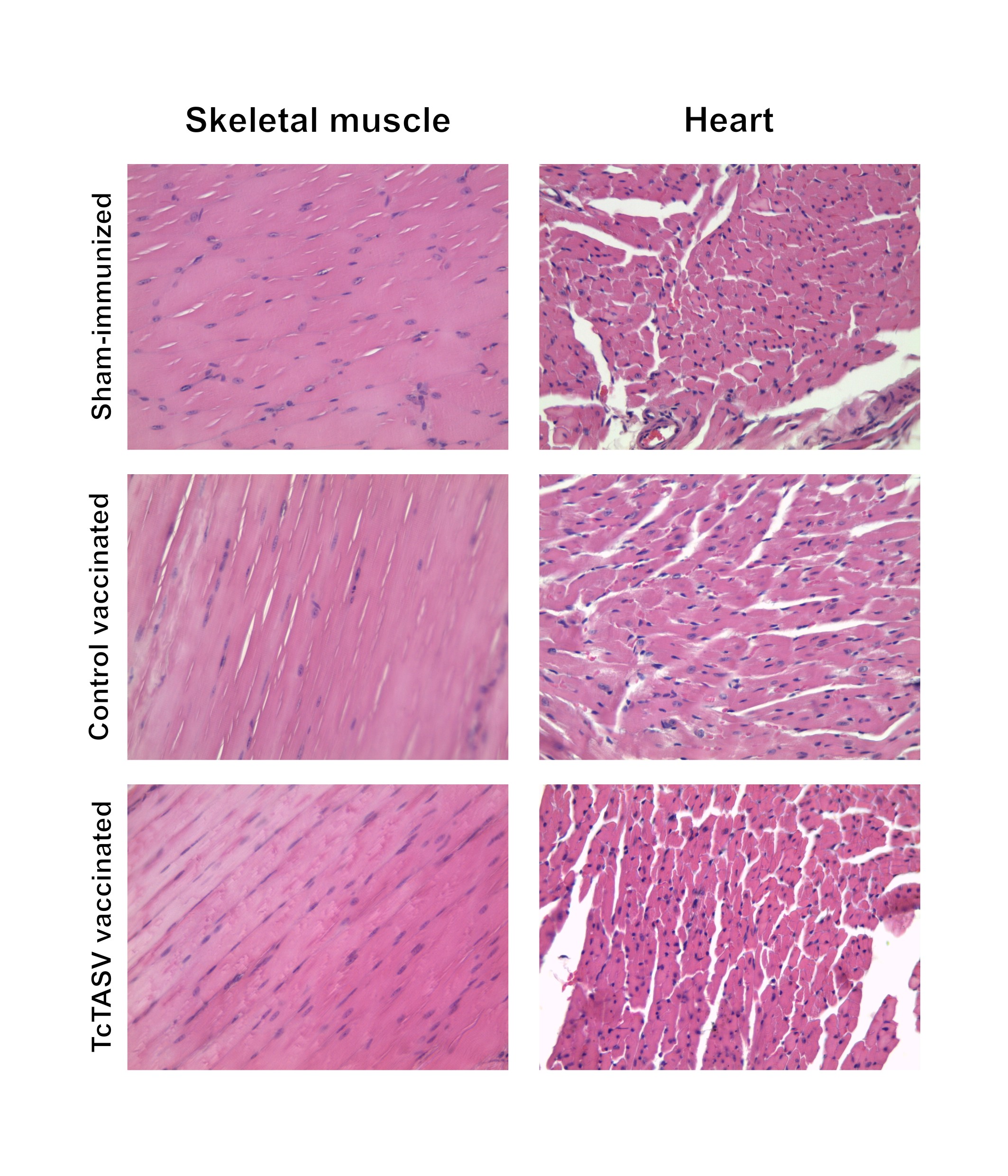

Supplement: Supplementary Figure 1 — Safety of the immunization protocol: Vaccination with rTcTASV-C and BV::ACAP does not affect the tissular architecture. Heart and skeletal muscles of TASV- and control-vaccinated, and sham-immunized mice were obtained 7 days after the last dose. Tissue sections were stained by H&E and analyzed under light microscopy. Magnification 100X. [file Image_1.jpeg]

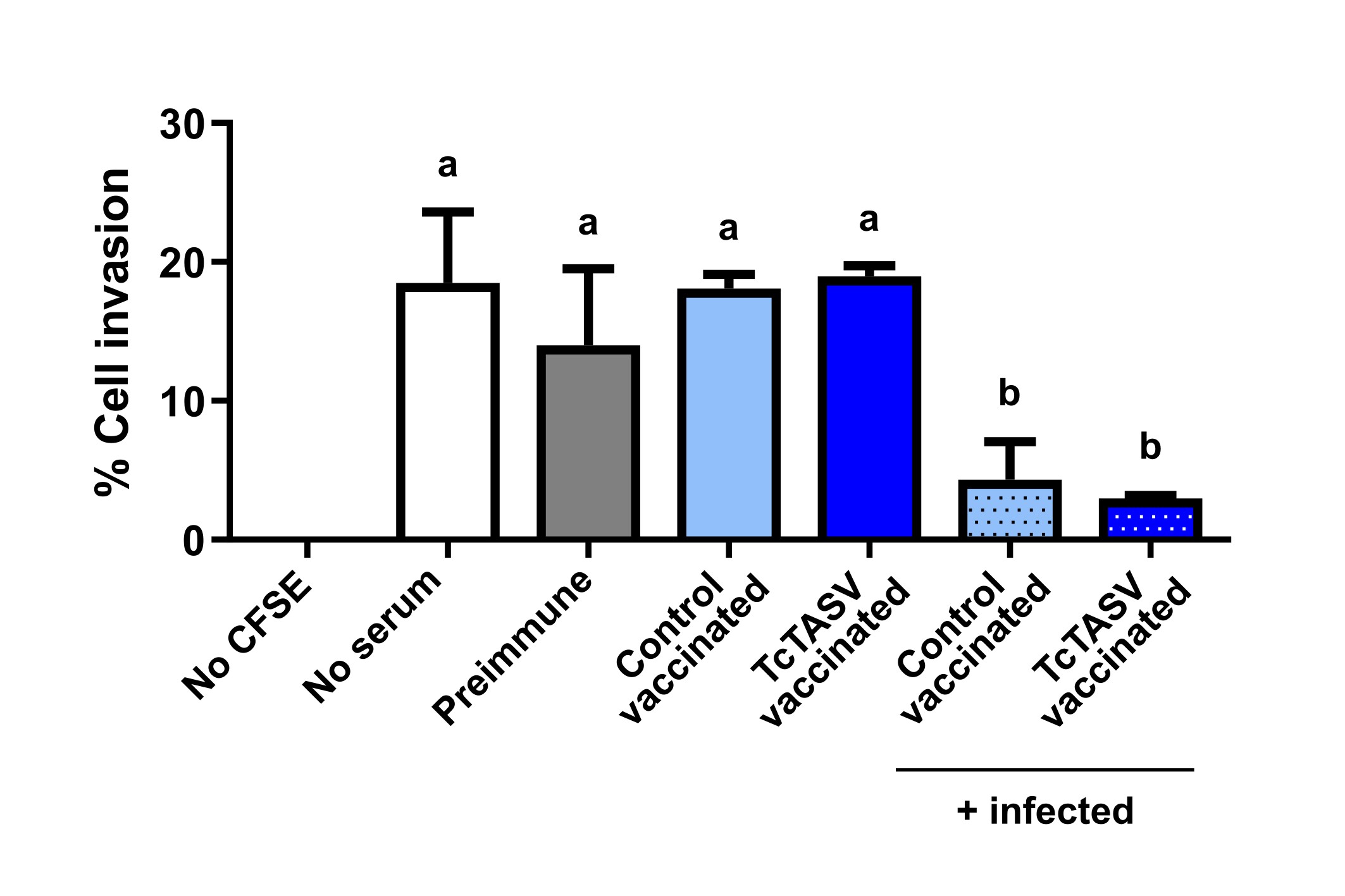

Supplement: Supplementary Figure 2 — Sera from TcTASV-vaccinated mice do not affect cellular invasion. CFSE-labeled trypomastigotes were incubated with sera from vaccinated, control or infected mice for 60 minutes and then used to infect Vero cells, as indicated in Section 2.7. The percentage of infected cells was determined at 24 h by flow cytometry. Ordinary one-way ANOVA plus Tukey’s post-test (group a vs group b: p ≤ 0.05). [file Image_2.jpeg]
